# Elective Neck Dissection during Salvage Total Laryngectomy: Personal Experience

**DOI:** 10.3390/jcm11051438

**Published:** 2022-03-05

**Authors:** Jacopo Galli, Giovanni Di Cintio, Stefano Settimi, Antonio Salvati, Claudio Parrilla, Giovanni Almadori, Gaetano Paludetti

**Affiliations:** 1 Unit of Otolaryngology—Head and Neck Surgery, “A. Gemelli” Hospital Foundation IRCCS, 00168 Rome, Italy; jacopo.galli@iol.it (J.G.); claudio.parrilla@policlinicogemelli.it (C.P.); giovanni.almadori@unicatt.it (G.A.); gaetano.paludetti@unicatt.it (G.P.); 2 Department of Head and Neck and Sensory Organs, Catholic University of the Sacred Hearth, 00168 Rome, Italy; 3 Unit of Otolaryngology, “Nuovo Ospedale Degli Infermi”, 13875 Ponderano, Italy; dicintiogiovanni@gmail.com; 4 Airway Surgery Unit, Pediatric Surgery Department, “Bambino Gesù” Children Hospital, 00165 Rome, Italy; drantoniosalvati@gmail.com

**Keywords:** elective neck dissection, larynx cancer, salvage total laryngectomy, occult nodal metastasis

## Abstract

The role of elective neck dissection during salvage surgery in patients with a clinically negative neck (cN0) is still discussed. The main objective of this work was to estimate the prevalence and predictive factors of occult neck nodes metastasis; we therefore aimed to evaluate the survival rate and the main oncologic outcomes of cN0 patients who underwent salvage total laryngectomy and elective bilateral neck dissection. In this retrospective observational study, we enrolled 80 cN0 patients affected by recurrent laryngeal cancer and who underwent salvage total laryngectomy and bilateral selective elective neck dissection. Several parameters were collected in order to find prognostic factors; finally, postoperative complications were reviewed and survival analysis was performed. Occult lymph node metastases were reported in 18 out of 80 patients (22.5%). Significant statistical correlation between lymphovascular invasion (*p* = 0.007), perineural invasion (*p* = 0.025) and occult nodal metastasis was found. Other variables (glottic subsite of recurrence, clinical T, pathological T, previous chemotherapy) were not significantly predictive of occult nodal metastasis. The 5-year OS, DSS, and RFS were 50.4%, 64.7%, and 63.4%, respectively. In conclusion, our single-institution data on a large cohort of patients, suggest performing routinely elective selective bilateral neck dissection during salvage total laryngectomy in cN0 patients due to the biological attitude of the tumor to spread to cervical nodes, considering an acceptable complications rate.

## 1. Introduction

Surgical and non-surgical organ preservation protocols markedly increased in the 3 last decades, even for locally advanced stages, inducing a relevant decrease of total laryngectomy as a primary treatment that on the contrary still represents the gold standard procedure after the failure of conservative approaches in case of locally recurrent or persistent laryngeal cancer. In this setting, the role of elective neck dissection (END) for clinically negative nodal metastasis (cN0) is still a challenge. Many authors do not recommend neck dissection during salvage surgery in cN0 patients, due to inconsistent data about the prevalence of occult neck metastasis, increased risk of complications, and a weak effect on locoregional control of the disease [1,2,3], while others suggest performing it due to the good impact on survival rate and for staging purposes as well [4], or in case of advanced recurrence stage (stage T3–T4) and supraglottic subsite of recurrence, because the rate of occult metastasis in these settings is higher [5,6]. As there are no conclusive data on this issue, including selective and superselective dissections, the management of these patients is often based on personal experience and practice patterns [7]. The aim of this study was at least to evaluate the role of neck dissection in cN0 patients who underwent salvage total laryngectomy, estimating the prevalence of pN+ and predictive factors for cervical lymph nodes metastasis. Secondary goals were the assessment of complication rate and the estimated five-year overall survival (OS), five-year relapse-free survival (RFS), and five-year disease-specific survival (DSS) in these patients.

## 2. Materials and Methods

### 2.1. Study Population and Study Design

This is a retrospective observational study of patients with recurrent laryngeal cancer and no evidence of neck node metastasis, who underwent salvage total laryngectomy and elective neck dissection at “A.Gemelli” University Hospital IRCCS in Rome, in a period from 2006 and 2019. Patients were classified as cN0 by combining the absence of cervical lymph nodes with increased consistency by clinical examination and the absence of lymph nodes with a diameter >1 cm by radiological evaluation. We included patients with recurrent laryngeal cancer after primary treatment failure (surgery, radiotherapy, or chemoradiotherapy) who underwent salvage total laryngectomy and bilateral selective elective neck dissection (levels II, III, IV ± VI). All patients underwent a full diagnostic workup, including a complete head and neck examination, videolaryngoscopy with narrow-band imaging examination, representative biopsy, chest CT, CT and/or MRI of the larynx and neck and Color-Doppler sonography of periaortic vessels and potential donor sites, if a free microvascular or pedicled rotation flap was planned. After workup, all cases were staged and discussed by the multidisciplinary tumor board, involving at least a radiologist, a medical oncologist, a radiation therapist, and a head and neck surgeon. The neoplasms were staged according to TNM 8 classification (tumor, node, metastasis) adopted by the American Joint Committee on Cancer (AJCC). Several parameters were collected: demographic information, history, recurrence subsite, clinical TNM of the recurrence, pathological TNM of the recurrence, histopathological findings like lymphovascular invasion and perineural invasion, postoperative complications, and survival.

### 2.2. Statistical Analysis

Statistical analysis was performed using IBM SPSS Statistics for Macintosh, Version 24.0. (IBM Corp., Chicago, IL, USA). Fischer’s Exact Test was performed in order to assess the correlation between clinical-histopathological characteristics of the tumor and the presence of occult neck node metastasis. The variables considered were the following: glottic subsite of recurrence (compared to supraglottic and transglottic together), clinical recurrence T, pathological recurrence T, perineural invasion, lymphovascular invasion, and previous chemotherapy. Finally, multiple logistic regression analysis was performed, in order to assess the association between the above-mentioned variables and the presence of occult neck node metastasis.

Survival curves were calculated by the Kaplan-Mayer method. We evaluated the 5-year OS, DSS, and RFS. The OS was the time from the salvage surgery to death resulting from any cause; the DSS was the time from salvage surgery to death specifically resulting from laryngeal cancer or to the last consultation for patients who were alive and well; the RFS was the time from salvage surgery to diagnosis of an eventual locoregional relapse.

## 3. Results

We analysed 80 patients (72 Males and 8 Females) with a mean age of 66 years (range, 42–86 years). The mean follow-up after salvage total laryngectomy was 41.89 ± 37.9 months (median: 56 months; range 4–150 months). At clinical staging 41 patients were classified as T4, 32 as T3, and 7 as T2. Pathological staging revealed 60 T4 patients, 17 T3, and only 3 T2. Regarding the site of recurrence, 26 patients had glottic recurrence, 29 patients had supraglottic recurrence, and 25 patients had trans-glottic recurrence. Cohort’s features are summarized in Table 1.

Occult lymph node metastases were reported in 18 (22.5%) out of 80 patients of which 7 in sovraglottic tumors, 7 in glottic ones, and 4 in transglottic ones. Furthermore, 8 occult metastases occurred in cT3 tumors and 10 in cT4 ones; instead, about the pathological extension of recurrence, they occurred in 3 pT3 tumors and in 15 pT4 ones. Regarding previous treatment, occult nodal metastasis occurred after primary surgery in 9/33 cases, after radiotherapy in 6/30 cases, and after chemoradiotherapy in 3/17 patients. 

At univariate analysis, a significant statistical correlation between lymphovascular invasion, perineural invasion, and occult nodal metastasis was found (*p* = 0.007 and *p* = 0.025 respectively). Other variables (glottic subsite of recurrence, clinical T, pathological T, previous chemotherapy) were not significantly predictive of occult nodal metastasis. 

At multiple logistic regression analysis as well, factors independently associated with occult nodal metastasis were lymphovascular invasion and perineural invasion, as shown in Table 2.

**Figure 1:**
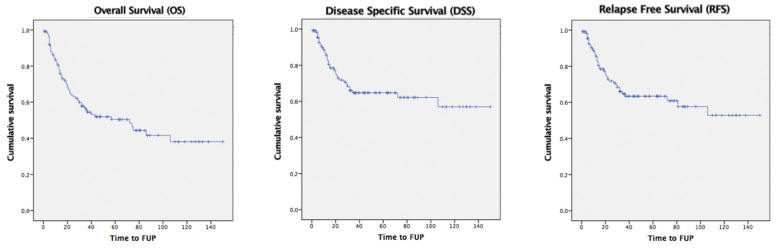
5-years **overall** survival (**left** panel), disease-specific survival (**middle** panel), and relapse free survival (**right** panel).

The 5-year OS, DSS, and RFS were 50.4%, 64.7%, and 63.4%, respectively, as shown in Figure 1. Post-operative complications are shown in Table 3.

## 4. Discussion

The role of elective neck dissection in cN0 patients during salvage total laryngectomy is still challenging for the surgeon, as there is no consensus about selection criteria for patient who could benefit from it as it is necessary to better balance the rate of occult nodal metastases with morbidity of the procedure.

Firstly, the rate of occult nodal metastasis from clinically N0 necks in patients receiving salvage laryngeal surgery varies greatly between studies. In the last years, in fact, several studies reported an incidence of occult cervical metastasis less than 10% [1,2,3,8,9,10], or between 10 and 20% [4,5,6,11,12,13,14,15,16], while only three studies recorded an incidence greater than 20% [7,17,18]. In particular, two main recent reviews on this topic published by Gross et al. and Lin et al. reported an occult nodal metastasis rate of 11% and 13.7%, respectively [19,20], significantly below 20%, which is widely considered the cut-off value to indicate or not an elective neck dissection in head and neck squamous cell carcinoma [21]. Instead, in our cohort we observed an occult nodal positivity rate higher than 20% (22.5%), suggesting a potential benefit for elective neck dissections in our patients that underwent salvage laryngectomy. 

Regarding the morbidity of neck dissection in patients submitted to salvage total laryngectomy, while several authors reported a higher fistula rate when neck dissection was performed at the time of salvage laryngectomy [3,9,22], others, like Bernard et al., reported a comparable complication rate between patients with and without END [8]. In our cohort, the overall complication rate after salvage total laryngectomy and simultaneous bilateral neck dissection was 45%, a noteworthy number, but at the inferior limit of the range reported in the literature, that ranges from 43% to 66% [3,14]. Concerning pharingocutaneous fistula, the most insidious complication after total laryngectomy, it occurred only in 18.75% of our patients, similarly to other case series of the literature. in which the incidence of fistula ranges from 10% to 16% after primary laryngectomy, and from 12% to 38% after salvage surgery, suggesting that in our series its incidence did not seem to be increased by neck dissection [22]. 

The impact on the survival of END in this setting is another controversial issue. Hilly et al. [4] reported a survival benefit in terms of DFS and OS for patients submitted to END, but only for recurrent T3/T4 and not for recurrent T1/T2. Our results about OS, DSS, and RFS in our population, are consistent with data reported by Hilly et al. [4], suggesting a possible improvement in survival rates and disease control with END. However, Lin [20] suggests that data about survival should be interpreted with caution because T-stage distribution between treated necks and observed neck is often not homogeneous, and many studies, which enrolled small numbers of patients, are underpowered to detect a survival benefit. In our study, the relationship between the tumor subsite and the risk of occult nodal metastasis was not statistically significant, whereas other authors found a significant correlation [5,6,7,13], suggesting END for supraglottic and transglottic recurrences compared to glottic ones. Although all the occult nodal metastasis occurred in locally advanced laryngeal recurrence (T3 and T4 stages) and none in the T2 stage, we did not find a significant difference with tumor extension. This result could be explained because of non-homogeneity of T stage distribution in our cohort (see Table 1), characterized by a little sample of T2 compared with T3 and T4. As well, primary treatment was not related to occult metastasis.

Interestingly, the relationship between pathological features, such as perineural invasion and lymphovascular invasion, and occult nodal metastasis was statistically significant, at multiple logistic regression analysis as well, suggesting their role as biological markers of aggressivity of the disease, so much that they could address adjuvant treatments in the last NCCN guidelines [23]. Particularly, perineural invasion is an important risk factor for local and regional recurrences and is considered an important independent predictor of survival of patients with squamous cell carcinoma of the head and neck [24,25,26]. In a recent manuscript, Fletcher et al. described that perineural invasion adversely affects survival and outcomes of patients who underwent salvage laryngectomy [27]. About lymphovascular invasion, Ylmaz et al. indicated that its presence significantly influences the disease-free survival and locoregional recurrence [28]. Considering that recurrent head and neck squamous cell carcinomas are aggressive malignancies with a high morbidity and mortality profile [29], as shown also by the high percentage of unfavorable pathologic findings such as lymphovascular invasion and perineural infiltration [30], the risk of occult nodal metastasis is remarkable in these patients. 

The strength of our study is that it is a single-center study, therefore it could guarantee homogeneity in surgical technique and patient care. In particular, every patient underwent bilateral elective neck dissection, which is always performed during total laryngectomy, both in a primary setting and in the salvage setting. On the contrary, the most important limitation of this study lies in its retrospective nature of the study, which could affect the completeness of our data. In fact, it would have been interesting to correlate the initial stage and clinical characteristics of the first tumor with the prevalence of occult metastasis, but in our series, we collected data about patients that we treated for a recurrence after RT/CT-RT failure, and in many cases, they came to our attention for the first time with the relapse. Finally, our data cannot demonstrate the impact of END over survival and prognosis, due to the absence of a control group treated only with salvage laryngectomy and clinical follow-up of the neck. Our findings, in terms of well-defined occult metastasis prevalence, good survival rates, and low incidence of complication after END, support all those authors who affirm the usefulness of elective neck dissection in salvage laryngeal surgery of cN0 patients. Further studies with larger populations are needed to clarify the superiority of this practice in terms of survival and disease control.

## 5. Conclusions

In conclusion, our data suggest performing routinely elective selective bilateral neck dissection during salvage total laryngectomy in cN0 patients due to its acceptable complications’ rate, not only for the biological attitude of head and neck squamous cell carcinoma to be aggressive and to easily spread to locoregional nodes but also for the high morbidity profile and high complications’ rate of a potential third surgical procedure in case of further neck recurrence.

## Figures and Tables

**Table 1:** Demographics and clinical characteristics of the case series.

Characteristics	Number of Patients (%)
Age at recurrence, years	
Median	66
Range	42–86
Sex	
Male	72 (90%)
Female	8 (10%)
Follow-up period, months	
Mean	41.89 ± 37.9
Range	1–150
Subsite	
Supraglottic	29 (36.25%)
Glottic	26 (32.5%)
Transglottic	25 (31.25%)
Previous Treatment (%)	
Radiotherapy + Chemotherapy	17 (21.25%)
Radiotherapy	30 (37.5%)
Surgery	33 (41.25%)
Clinical T classification (%)	
T2	7 (8.75%)
T3	32 (40%)
T4	41 (51.25%)
Pathological T classification (%)	
T2	3 (3.75%)
T3	17 (21.25%)
T4	60 (75%)
Pathological N classification (%)	
Positive	18 (22.5%)
Negative	62 (77.5%)

**Table 2:** Results of multiple logistic regression analysis.

	Odds Ratio	*p*	95% CI
Glottic subsite	0.451	0.09	0.180–1.133
Previous chemotherapy	0.601	0.16	0.274–2.618
Perineural invasion	6.413	**0.014**	1.455–28.274
Lymphovascular invasion	6.293	**0.032**	1.174–33.725
cT	1.103	0.88	0.296–4.110
pT	1.237	0.80	0.244–6.283

Abbreviations. CI: confidence interval.

**Table 3:** Complication’s rate of salvage total laryngectomy and elective bilateral neck dissection.

Type of Complication	Rate (%)
Pharyngoutaneous fistula	18.75%
Skin dehiscence	6.25%
Infections	5%
Hemorrhage	1.25%
Dysphagia	7.5%
Nerve XI Weakness	6.25%
Chyle leak	0%

## Data Availability

The data presented in this study are available on reasonable request from the corresponding author.
